# Anaphylaxis Triggered by Benzyl Benzoate in a Preparation of Depot Testosterone Undecanoate

**DOI:** 10.1155/2012/384054

**Published:** 2012-01-05

**Authors:** Gregory S. Y. Ong, Colin P. Somerville, Timothy W. Jones, John P. Walsh

**Affiliations:** ^1^Department of Endocrinology and Diabetes, Sir Charles Gairdner Hospital, 1st Floor, C-Block, Hospital Avenue, Nedlands, WA 6009, Australia; ^2^The Allergy Centre, 64 Farrington Road, Leeming, WA 6149, Australia; ^3^School of Paediatrics and Child Health, University of Western Australia, GPO Box D184, Perth, WA 6840, Australia

## Abstract

We report the first case of an anaphylactic reaction to Reandron 1000 (depot testosterone undecanoate with a castor oil and benzyl benzoate vehicle). While considered to have a favourable safety profile, serious complications such as oil embolism and anaphylaxis can occur. In our patient, skin testing identified benzyl benzoate to be the trigger, with no reaction to castor oil or testosterone undecanoate components. As benzyl benzoate exists in multiple pharmaceuticals, foods, and cosmetics, individual components of pharmaceuticals should be tested when investigating drug allergies. Doctors should be alert to the potential for serious reactions to any of the components of Reandron 1000.

## 1. Introduction

In men requiring testosterone therapy, depot testosterone undecanoate (TU) is a useful option. Compared to conventional testosterone esters, depot TU maintains adequate testosterone levels with less frequent injections and has better pharmacokinetics. Specifically, depot TU comparatively achieves higher trough serum testosterone concentrations without the wide variation between peak and trough levels between doses [1]. TU was initially developed in the 1970s as an oral testosterone replacement preparation, with a transdermal patch available in the 1990s [1]. In China, a depot TU preparation with a Chinese teaseed oil vehicle was manufactured for intramuscular use and found to have a long half-life of 21 days, longer than conventionally used testosterone esters [2, 3]. Depot TU is currently marketed with a castor oil and benzyl benzoate vehicle [4], as the castor oil affords an even longer half-life (33.9 days) than the original Chinese depot preparation [2, 5]. 

The long-term overall safety profile of depot TU has been generally favourable [6, 7]. Anaphylaxis to depot TU has not been specifically reported although hypersensitivity is listed as an uncommon adverse effect in the manufacturer's product information [4]. Hypersensitivity reactions have not been described in cohorts who have used this preparation from 4 to 8 years. 

Here, we report the first documented case of anaphylaxis to Reandron 1000, a depot preparation of TU. This case is notable for the fact that the responsible agent was not the main active ingredient.

## 2. Case Presentation

A 16-year-old boy with primary hypogonadism due to bilaterally absent testes, but otherwise without remarkable medical history, was converted from monthly intramuscular injections of testosterone esters (Sustanon, Schering-Plough) to depot testosterone undecanoate (Reandron 1000, Bayer) due to debilitating fluctuations in mood and energy levels. There was significant improvement in symptoms on the depot preparation.

Within four minutes after his third dose was administered, he developed sweatiness, facial swelling, itching, urticaria, and sensation of throat obstruction with chest tightness. He was normotensive without tachycardia. He was treated with intravenous promethazine and hydrocortisone 250 mg and observed in an emergency department. Adrenaline (epinephrine) was not administered. The differential diagnosis of oil embolism was not pursued in view of the classical clinical features of anaphylaxis.

A skin prick test found definite reaction to Reandron 1000 with a 10 × 8 mm wheal, but no reaction to testosterone esters gel or saline solution control. Testing of the components of Reandron 1000 found that non-skin-irritating concentrations of benzyl benzoate resulted in a 10 × 10 mm wheal and smaller peripheral lesions. Neither castor oil nor TU induced a response. His father was tested as a control and did not react to any of the components.

Since discontinuation of Reandron 1000, the patient has used topical testosterone ester gel and crystalline testosterone pellets were implanted subcutaneously. There have been no further episodes of anaphylaxis.

## 3. Discussion

We report a case of anaphylaxis to a depot preparation of TU comprising three components. Testing of each component identified benzyl benzoate as the likely trigger and demonstrates the importance of testing every component when investigating and managing medication-related anaphylaxis. Neither Reandron 1000 nor benzyl benzoate has been previously reported as a trigger for anaphylaxis.

Excipients are the components of pharmaceuticals apart from the active substance. They fulfil a variety of different roles including colouring, flavouring, and alteration of the stability, solubility, durability, or permeability of the active ingredient. These agents are capable of inducing severe adverse drug reactions, particularly in the paediatric population [8]. Immunologically mediated hypersensitivity reactions can manifest as immediate (within 1 hour) onset of urticaria, angioedema, or anaphylaxis. Alternatively, there can be a delayed (>1 hour) onset of mostly cutaneous symptoms which, as in Stevens-Johnson syndrome, can still be severe [9]. In Reandron 1000, the excipient agents are benzyl benzoate and castor oil, while the active ingredient is TU.

Benzyl benzoate (chemical formula C_14_H_12_O_2_) is a colourless oily liquid which is rapidly metabolised by the body to benzoic acid and benzyl alcohol [10]. The agent is widely used as an additive in many different pharmaceutical and nonpharmaceutical products for human consumption. For instance, it is used as a preservative, a solvent in perfumes, a flavouring agent in foods and medications, and in insecticides and insect repellents [10, 11]. In oil-based vehicles for depot steroids, it lowers viscosity to improve ease of administration [12] and prevents crystallisation of steroids during storage [10, 13]. In testosterone preparations, it is also found in testosterone cypionate (Depo-Testosterone, Pharmacia) but not testosterone esters (Sustanon) or testosterone gel.

 In its role as a topical insecticide for scabies, benzyl benzoate is applied in a diluted solution with a concentration between 10% and 25%. It is known to cause skin irritation and contact dermatitis in this context, but hypersensitivity reactions have not been recorded in existing studies. No information is available as to whether or not benzyl benzoate possesses the intrinsic ability to induce mast cell degranulation. Databases for adverse reactions have also identified convulsions occurring with ingested benzyl benzoate [10, 14].

Although not previously reported, hypersensitivity reactions to benzyl benzoate are unlikely to be isolated to our patient. Its presence in numerous consumer products raises the possibility of underreporting due to lack of awareness and failure to identify it as the trigger. Existing guidelines by the British Society for Allergy and Clinical Immunology [15] recommend skin prick testing if a compatible history of IgE-mediated drug hypersensitivity exists. In our patient, signs and symptoms of anaphylaxis to testosterone therapy and the clinical need for ongoing testosterone therapy warranted such investigation. Although intuitive and in common practice, there are no specific recommendations regarding testing of individual components of pharmaceutical preparations in either British or Australian [16] guidelines. In our patient, discovery of a reaction to a single component of the depot vehicle will better guide selection of testosterone replacement therapies. It will also allow him to remain vigilant to benzyl-benzoate-containing products.

Castor oil has been used as a vehicle for steroid hormones for decades, prolonging their effect compared to equivalent aqueous suspensions by increasing storage in fatty depots in the body [12]. While there have been no reports of anaphylaxis to castor oil, sudden onset of respiratory symptoms can signify oil-related pulmonary microembolism after entry via the lymphatic or venous system. This complication is rare [17].

In this case, our patient received supportive care for anaphylaxis with antihistamines and glucocorticoids. The immediate management of anaphylaxis is not guided by randomised placebo-controlled trials which are unethical due to the potential for rapid progression to fatality arising from delay of treatment. The use of adrenaline (epinephrine) is widespread and recommended in multiple guidelines as first-line therapy based upon results of uncontrolled studies [18]. However, recommendations for use of adjunctive agents such as antihistamines and glucocorticoids are more heterogeneous. The use of H1-antagonists as a first-line agent is not recommended, as they are of slow onset, fail to relieve bronchospasm or gastrointestinal symptoms, and have anticholinergic effects which can induce drowsiness and confuse the clinical picture in a critically ill patient [19]. Similarly, glucocorticoids do not acutely relieve symptoms but prevent prolonged or biphasic symptoms of anaphylaxis [18]. These agents are best used in conjunction with, rather than in place of adrenaline.

## 4. Conclusion

Anaphylactic reactions can occur to the benzyl benzoate component of depot preparations of testosterone undecanoate. It is potentially unrecognised or can be misdiagnosed as oil embolism and underreported. Testing for reactions to the individual components of pharmaceutical agents may prevent inappropriate exclusion of all available preparations of a particular agent if it is the vehicle rather than the active ingredient that is causative. Similarly, it will alert the patient to risk of hypersensitivity to unrelated products which may utilise the same agent. Doctors should remain aware of the potential for serious reactions to testosterone replacement therapies and should consider appropriate further investigation.
